# Documenting cannabis use in primary care: a descriptive cross-sectional study using electronic medical record data in Alberta, Canada

**DOI:** 10.1186/s13104-023-06274-6

**Published:** 2023-02-01

**Authors:** Boglarka Soos, Stephanie Garies, Ashley Cornect-Benoit, Lori Montgomery, Heather Sharpe, Katherine Rittenbach, Donna Manca, Kimberley Duerksen, Brian Forst, Neil Drummond

**Affiliations:** 1 Department of Family Medicine, G012 Health Sciences Centre, University of Calgary, 3330 Hospital Drive NW, Calgary, AB T2N 4N1 Canada; 2 Department of Community Health Sciences, University of Calgary, Calgary, AB Canada; 3 Department of Anesthesiology, Perioperative and Pain Medicine, University of Calgary, Calgary, AB Canada; 4 Department of Medicine, University of Alberta, Edmonton, AB Canada; 5 Department of Medicine, University of Calgary, Calgary, AB Canada; 6 Department of Psychiatry, University of Calgary, Calgary, AB Canada; 7 Department of Psychiatry, University of Alberta, Edmonton, AB Canada; 8 Department of Family Medicine, University of Alberta, Edmonton, AB Canada

**Keywords:** Primary care, Cannabis, Electronic medical records, Data quality

## Abstract

**Objective:**

Documenting cannabis use is important for patient care, but no formal requirements for consistent reporting exist in primary care. The objective of this study was to understand how cannabis use is documented in primary care electronic medical record (EMR) data.

**Results:**

This was a cross-sectional study using de-identified EMR data from over 398,000 patients and 333 primary care providers in Alberta, Canada. An automated pattern-matching algorithm was developed to identify text and ICD-9 diagnostic codes indicating cannabis use in the EMR. There was a total of 11,724 records indicating cannabis use from 4652 patients, representing approximately 1.2% of the patient sample. Commonly used terms and ICD-9 codes included *cannabis*, *marijuana/marihuana, THC,* 304.3 and 305.2. Nabilone was the most frequently prescribed cannabinoid medication. Slightly more males and those with a chronic condition had cannabis use recorded more often. Overall, very few patients have cannabis use recorded in primary care EMR data and this is not captured in a systematic way. We propose several strategies to improve the documentation of cannabis use to facilitate more effective clinical care, research, and surveillance.

**Supplementary Information:**

The online version contains supplementary material available at 10.1186/s13104-023-06274-6.

## Introduction

Cannabis is an unusual substance as it is both the most commonly used non-medical psychoactive substance in Canada [1] and is also used medically, which can complicate interpretation of its function. A 2017 national survey reported that 15% of Canadians had used cannabis in the previous year, up from 12% in 2015 [1]. Higher rates were observed in youth aged 15–19 years (19%) and young adults aged 20–24 years (33%) compared to adults 25 years of age and older (13%) [1]. Although cannabis has been accessible in Canada for medical purposes since 2001 [2], the federal government enacted the *Cannabis Act* in 2018, which legalized limited non-medical possession and use of cannabis [3].

Primary care is usually the first point of entry into the Canadian healthcare system and is the setting for many preventative activities, including understanding patient use of alcohol, tobacco, and drugs. It has been suggested that primary care providers ask all patients about their use of cannabis at least once, in order to assess for potential drug interactions, provide education on the possible risks and harms, propose more effective alternative treatment if applicable, ensure safety when engaging in use, and screen for unhealthy use [4–7]. Unfortunately, many electronic medical record (EMR) systems are largely ill-equipped to capture this information in structured and searchable fields. Further, there is no agreed-upon guideline for documenting cannabis use in the EMR, including the systematic collection of method, dose, and frequency. The standardized capture of cannabis use information would serve two purposes: to have a record in the EMR that can be easily referenced by primary care team members for appropriate patient care, including monitoring for misuse and possible side effects; and for secondary purposes, such as cannabis-related research and public health surveillance. The goal of this study is to understand the documentation patterns for patient cannabis use observed in primary care EMR data.

## Main text

### Data source

This was a cross-sectional study using EMR data from Alberta, Canada, which was collected by the Canadian Primary Care Sentinel Surveillance Network (CPCSSN). CPCSSN is comprised of 12 primary care practice-based research networks who organize the regional collection of de-identified patient-level data from the EMR systems of participating family physicians and nurse practitioners [6]. The data are extracted, cleaned, and processed twice per year and include patient demographics, diagnoses, medications, laboratory results, physical measurements (e.g. blood pressure, height, weight), behavioural risk factors, and physician billing claims [6]. Two regional networks contribute to CPCSSN in Alberta—the Northern and Southern Alberta Primary Care Research Networks (NAPCReN and SAPCReN, respectively). Between these two networks, EMR data were captured for over 430,000 patients from 333 providers in 55 clinics in 2019. This represent approximately 10% of patients and 6% of family physicians in Alberta [8, 9]. In relation to the rest of Canada, Alberta tends to have a slightly younger population (average age of 39 years compared to 41.9 in Canada) and higher median employment income in 2020 among full time workers ($70,500 in Alberta versus $63,600 in Canada) [10].

### Study sample

De-identified EMR data from patients in the most recent Alberta extraction were included. Better data quality is generally found in 2010 and later; therefore, all data between January 1, 2010 and June 30, 2019 (the date of the most recent data extraction at the time of this study) were used. However, the start date of a patient record can depend on when the practice first implemented an EMR system and when a patient first attended the clinic. The sample represents a variety of patients, providers, locations, and types of EMR systems across the province. In general, the CPCSSN data over-represents older adults and females, as is common in primary care contexts [11].

### Search strategy & analysis

The initial list of search terms related to cannabis use (including text words, International Classification of Diseases 9th Revision [ICD-9] codes, medication names, and medication codes using the Anatomical Therapeutic Chemical [ATC] Classification system) was developed by the study team, then circulated to additional primary care providers, health professionals, and researchers for further refinement. A regular expression-based approach was used to develop the search pattern based on this list, then applied to the database and reviewed for accuracy. Terms that did not generate any results or matched an unrelated entry (e.g., *grass allergy* or *breast bud*) were excluded (Additional file 2: Table S1). Specific inclusion and exclusion criteria were created according to the entries resulting from the search. This process resulted in the final, automated pattern-matching algorithm that was applied to the database.

Records that contained at least one indication of cannabis use based on the final search strategy were included. The type and frequency of records and terms were described by location in the EMR (i.e., table, field). The presence of a cannabis record was reported by patient characteristics such as gender, age group, urban or rural residence, and presence of chronic condition(s), as well as by type of EMR system. Data management and analysis were conducted using Python 3.7.3, StataSE 16.0, and R v4.2.1. This study was approved by the Conjoint Health Research Ethics Board at the University of Calgary (REB19-0429) and the Health Research Ethics Board at the University of Alberta (Pro00093062).

### Results

There were 11,724 records indicating cannabis use from a total of 4652 unique patients. Table 1 details the terms and ICD-9 codes representing cannabis use found in the EMR (additional details in Additional file 2: Table S1). In the Billing table (where codes are used for the submission of billing claims to government for payment, with ICD-9 required for diagnostic codes), ICD-9 codes 304.3x *(cannabis dependence)* and 305.2x *(non-dependent cannabis abuse)* were used to reflect a more problematic type of use. These codes were also used throughout other sections of the EMR, including Encounter Diagnosis (which consists of diagnoses, symptoms, or activities recorded during the patient visit or encounter), Family History, and Problem List (a list of the patients’ most significant health concerns). The most common text terms and variations of these terms were *cannabis*, *marijuana/marihuana,* and *THC.* These terms were found in all EMR tables except for Billing. Entries often included more than one term. A total of 8 different terms for non-medical cannabis were found, with an additional 5 terms identified referring to medical uses in the Medication table (Nabilone being the most common). These tables serve different purposes in the EMR; the terms/codes listed may have been recorded at any time during a routine visit, for billing purposes, or captured as part of the initial patient history.

**Table 1:** Cannabis-related terms and codes found in various sections of the EMR data

Sections of EMR data (*n* cannabis entries/*N* total entries in table)^a^	Term found in record (text or ICD-9 code)	Number (%) of records N = 11 724	Number (%) of patients N = 4 652
Billing (1 139/8 406 354)	304.3	621 (54.5)	373 (53.7)
	305.2	518 (45.5)	358 (51.6)
Encounter diagnosis (3 572/7 996 932)	Cann*b	1 930 (54.0)	989 (56.4)
	Marij OR Marih	1 692 (47.4)	910 (51.9)
	304.3	826 (23.1)	493 (28.1)
	305.2	668 (18.7)	437 (24.9)
	THC	191 (5.3)	150 (8.6)
	MJ	132 (3.7)	91 (5.2)
	CBD	72 (2.0)	53 (3.0)
	Pot	47 (1.3)	44 (2.5)
	Weed	12 (0.3)	12 (0.7)
	Hash	1 (< 0.1)	1 (0.1)
Family history (123 / 378 374)	Marij OR Marih	78 (63.4)	76 (62.8)
	Weed	31 (25.2)	31 (25.6)
	Cann*b	17 (13.8)	16 (13.2)
	304.3	5 (4.1)	4 (3.3)
	305.2	5 (4.1)	5 (4.1)
	MJ	2 (1.6)	2 (1.7)
	Pot	2 (1.6)	2 (1.7)
	CBD	1 (0.8)	1 (0.8)
	THC	1 (0.8)	1 (0.8)
Problem list (1 068 / 897 892)	Cann*b	649 (60.8)	638 (61.8)
	Marij OR Marih	643 (60.2)	628 (60.9)
	304.3	324 (30.3)	320 (31.0)
	305.2	232 (21.7)	230 (22.3)
	Pot	37 (3.5)	32 (3.1)
	MJ	26 (2.4)	24 (2.3)
	THC	17 (1.6)	17 (1.6)
	CBD	9 (0.8)	9 (0.9)
	Weed	4 (0.4)	4 (0.4)
Medication (5 210 / 7 009 108)	Nabilone	4 769 (91.5)	1 330 (80.9)
	Cann*b	183 (3.5)	151 (9.2)
	Marij OR Marih	157 (3.0)	137 (8.3)
	N02BG10	92 (1.8)	71 (4.3)
	CBD	62 (1.2)	54 (3.3)
	Sativex	37 (0.7)	25 (1.5)
	THC	21 (0.4)	17 (1.0)
	Marinol	9 (0.2)	5 (0.3)
	Sativa	8 (0.2)	8 (0.5)
Risk factor (612 / 554 727)	Marij OR Marih	238 (38.9)	230 (39.0)
	THC	219 (35.8)	219 (37.2)
	MJ	61 (10.0)	57 (9.7)
	Cann*b	48 (7.8)	43 (7.3)
	Pot	26 (4.2)	26 (4.4)
	Weed	18 (2.9)	18 (3.1)
	CBD	4 (0.7)	4 (0.7)
	Hash	2 (0.3)	2 (0.3)

*patients can have more than one entry in their EMR

Table 2 describes the characteristics of patients who are in the Alberta CPCSSN data and who had at least one record of cannabis use in their EMR. Overall, there was a relatively low frequency of cannabis use recorded. Male patients (n = 2661) had slightly more total records of cannabis use compared to females (n = 1990). Youth 19 years and younger had the lowest proportion of cannabis use documented in their EMR. Among those with a record of cannabis use, over half had depression (66% in females, 51% in males), roughly a quarter had hypertension (27% in females, 25% in males), and many had osteoarthritis (23% in females, 18% in males).

**Table 2:** Characteristics of patients with a cannabis term recorded in their primary care electronic medical record

Patient characteristics	All patients in the Alberta EMR Database, n (%)		Patients with Cannabis use recorded in their EMR, n (%)		P-Value
	Females N = 212 819	Males N = 185 711	Females N = 1 990	Males N = 2 661	
Age group in years					p < 0.001
Under 10	21 349 (10.0)	23 544 (12.7)	5 (0.3)	7 (0.3)	
10–19	22 265 (10.5)	21 579 (11.6)	68 (3.4)	88 (3.3)	
20–29	26 004 (12.2)	19 902 (10.7)	380 (19.1)	483 (18.2)	
30–39	33 669 (15.8)	24 555 (13.2)	389 (19.5)	493 (18.5)	
40–49	28 026 (13.2)	24 643 (13.3)	322 (16.2)	442 (16.6)	
50–59	28 252 (13.3)	25 597 (13.8)	390 (19.6)	544 (20.4)	
60–69	24 993 (11.7)	23 247 (12.5)	257 (12.9)	426 (16.0)	
70 and older	28 261 (13.3)	22 644 (12.2)	179 (9.0)	178 (6.7)	
Residence location					p < 0.001
Rural	37 812 (17.8)	33 514 (18.0)	377 (18.9)	395 (14.8)	
Urban	167 246 (78.6)	144 230 (77.7)	1 501 (75.4)	2 028 (76.2)	
Missing postal code	7 761 (3.6)	7 967 (4.3)	112 (5.6)	238 (8.9)	
Presence of chronic conditions as defined by CPCSSN algorithms					
CKD	9 450 (4.4)	6 752 (3.6)	132 (6.6)	87 (3.3)	p < 0.001
COPD	6 999 (3.3)	7 735 (4.2)	206 (10.4)	382 (14.4)	p < 0.001
Depression	51 368 (24.1)	27 695 (14.9)	1315 (66.1)	1350 (50.7)	p < 0.001
Diabetes mellitus	15 564 (7.3)	16 468 (8.9)	267 (13.4)	289 (10.9)	p < 0.001
Epilepsy	2 664 (1.3)	2 635 (1.4)	83 (4.2)	120 (4.5)	p < 0.001
Hypertension	38 374 (18.0)	35 441 (19.1)	534 (26.8)	652 (24.5)	p < 0.001
Osteoarthritis	24 602 (11.6)	17 058 (9.2)	453 (22.8)	482 (18.1)	p < 0.001
Parkinson’s disease	477 (0.2)	622 (0.3)	9 (0.5)	22 (0.8)	p < 0.001
Pediatric asthma	8 990 (4.2)	10 538 (5.7)	70 (3.5)	77 (2.9)	p < 0.001
None^a^	112 299 (52.8)	103 876 (55.9)	384 (19.3)	720 (27.1)	p < 0.001

*CKD*   Chronic kidney disease; *COPD* Chronic obstructive pulmonary disease; *CPCSSN* Canadian Primary Care Sentinel Surveillance Network; *EMR*  Electronic medical record

^a^no co-morbid conditions from 13 conditions that have a CPCSSN validated case definition

We examined the estimated time period between when the first cannabis record appeared in a patient’s EMR (from 2010 onward) and the number of years of information present in that patient's record (Additional file 1: Figure S1). Most patients had cannabis used documented within their first 5 years of data, either beginning at first appointment within the practice or when the EMR was implemented in the clinic. There was an observable increase in cannabis recording from late 2015 onwards, perhaps reflecting increasing public discourse around the potential legalization of cannabis in Canada.

Cannabis documentation was also examined by type of EMR system (Additional file 2: Table S2). Less than 2% of all patients had a record of cannabis use, but this varied by EMR from 1.0% to 1.7% (p < 0.001). We also investigated differences in cannabis use documentation according to provider characteristics (e.g. age, sex, urban or rural location of practice), however no differences in these characteristics were detected.

### Discussion

Overall a very small proportion of patients had cannabis use documented, especially compared to prevalence estimates in the general population [12]. This is consistent with other types of behavioural risk factor data, such as alcohol use or smoking, which tend to be missing often or not recorded in a structured or easily extractable way in primary care EMRs [13–16]. Relative to all patients within the Alberta CPCSSN database, patients with a record of cannabis use were more likely to have a chronic condition. These findings may suggest an informed presence bias [17], where patients seeking care more frequently tend to have more opportunities for discussions around current substance use or screening. Further, these findings may indicate the use of cannabis as an alternative, patient-initiated treatment option for chronic conditions such as depression; Rotermann et al. found that cannabis use for some self-defined medical purposes in addition to non-medical purposes was higher among Canadians who reported fair to poor physical or mental health compared to those in better general or mental health [18].

The lack of observed cannabis use records in the EMR data may be due to multiple factors—providers may not ask all patients about cannabis use, patients may be unwilling to disclose it due to persisting stigma around social acceptability, or this information may be recorded in areas of the EMR that CPCSSN is unable to extract from (e.g. narrative notes). Alternatively, perhaps a patient-provider discussion of cannabis use occurred but was not recorded, particularly prior to its legalization [6, 7, 12]. Various text terms were used but without specific information related to amount, frequency, or method, which is necessary to help clinicians evaluate potential drug-drug interactions or to monitor high-risk use. Lastly, there is insufficient information to indicate if the cannabis is medically indicated or used for non-medical purposes. While cannabinoid medications with a Drug Information Number (DIN) will be recorded similarly to other types of medication in the EMR, herbal cannabis does not have a DIN and, until 2018, was subject to additional documentation required by Health Canada and regulatory bodies (which would not be part of the CPCSSN database). After legalization in Canada, patients can now obtain cannabis from retail sources without a prescription from a healthcare provider, although many still report using it for medical purposes; this information would also not be included as medication data in the CPCSSN database unless disclosed by the patient. However, a recent Canadian survey suggests that most cannabis is purchased from either legal storefronts or a legal online source (approximately 54%) and very few respondents reported obtaining a medical document from a healthcare provider (an estimated 3% of Canadians) [19], meaning that the EMR database is likely missing most uses of cannabis apart from instances obtained through medical professionals (which appear to make up a very small proportion of cannabis use) or when elicited and recorded by physicians during clinical visits. The overall lack of cannabis use information accessible in our primary care EMR database presents challenges not only for secondary uses but may also potentially contribute to gaps in clinical care.

### Suggestions to improve cannabis documentation

Strategies to improve cannabis use documentation are multi-faceted. This largely relies on having adequate resources, support and time in busy primary care practices. Nevertheless, it is important for clinicians to understand what substances (including drugs and over-the-counter medications) are being taken or used by their patients. For patients who indicate that they are using cannabis, we suggest the following additional information be collected [20]:

1. Age at initiation;
2. Current frequency of use, with quantities (e.g., once per week)
3. Method of use (e.g., vaporized, smoked, synthetics, oils, etc.)
4. How much is used or consumed (in milligrams or grams, if known; THC and CBD content, if known);
5. Reason for use;
6. Reported side effects, if any.

Another critical aspect is ensuring that EMR systems offer easy-to-use, structured fields to capture this information, as well as an area with tools available to assess unhealthy use of cannabis (e.g., Alcohol, Smoking and Substance Involvement Screening Test [ASSIST], Cannabis Use Disorder Identification Test-Revised [CUDIT-R]). Although a minimum data set for Canadian primary care EMRs has been developed, it does not include details about risk factors and only recommends capturing the date of onset and a code representing “social behaviour that increases the possibility of disease or injury” or problematic use [21]. This minimum data set is not mandatory and a wide variety of data elements and quality can be observed in multi-jurisdictional EMR data, largely due to the many different types of EMR products available in Canada [16, 22–24].

## Limitations

To our knowledge, this study was the first in Canada to examine how cannabis use is being documented in primary care EMRs. However, the analysis was limited to one province and to a subset of providers and patients contributing to CPCSSN in Alberta. Moreover, not all data in the EMR were available for analysis, as CPCSSN data in Alberta does not include narrative notes or PDF documents. There also may be additional terms or codes reflecting cannabis use that were too generic to include here (e.g., ‘substance use’).

The systematic and consistent recording of cannabis use in primary care is important for comprehensive patient care and improving our knowledge about trends in cannabis use. This study described large inconsistencies and gaps in recording this information. EMR vendors should prioritize offering a dedicated and structured area in the EMR to capture cannabis and other substance use data to assist clinicians with capturing higher quality, more consistent, and clinically meaningful EMR data for patient care and practice evaluation and improvement.

## Supplementary Information

**Additional file 1: Figure S1.** Length of time to the first mention of cannabis in the patient record (from earliest date in the EMR).

**Additional file 2: Table S1.** Search terms used in the EMR data.

**Additional file 3: Table S2.** Differences in cannabis-related recording by type of EMR system.

**Additional file 4: Table S3.** Characteristics of patients with a cannabis term recorded in their primary care electronic medical record, stratified by presence of record in the Medication table.

## Data Availability

The de-identified datasets supporting the conclusions of this article are available as two separate datasets through the respective regional networks in Alberta: NAPCReN, http://napcren.ca; SAPCReN, http://sapcren.ca. Data access procedures and requirements vary by network; for more information, please contact the corresponding author or visit the aforementioned websites.

## Abbreviations

ASSIST: Alcohol Smoking and Substance Involvement Screening Test
ATC: Anatomical Therapeutic Chemical (classification system)
CBD: Cannabidiol
CPCSSN: Canadian Primary Care Sentinel Surveillance Network
CUDIT-R: Cannabis Use Disorder Identification Test-Revised
DIN: Drug Information Number
EMR: Electronic Medical Record
ICD-9: International Classification of Diseases, 9th revision
NAPCReN: Northern Alberta Primary Care Research Network
SAPCReN: Southern Alberta Primary Care Research Network
THC: Tetrahydrocannabinol
