# Supplementary material for: Documenting cannabis use in primary care: a descriptive cross-sectional study using electronic medical record data in Alberta, Canada
Source: BMC Res Notes. 2023 Feb 1;16:9. doi: 10.1186/s13104-023-06274-6 (PMC9890680; doi:10.1186/s13104-023-06274-6)

**Figure S1**. Length of time to the first mention of cannabis in the patient record (from earliest date in the EMR).


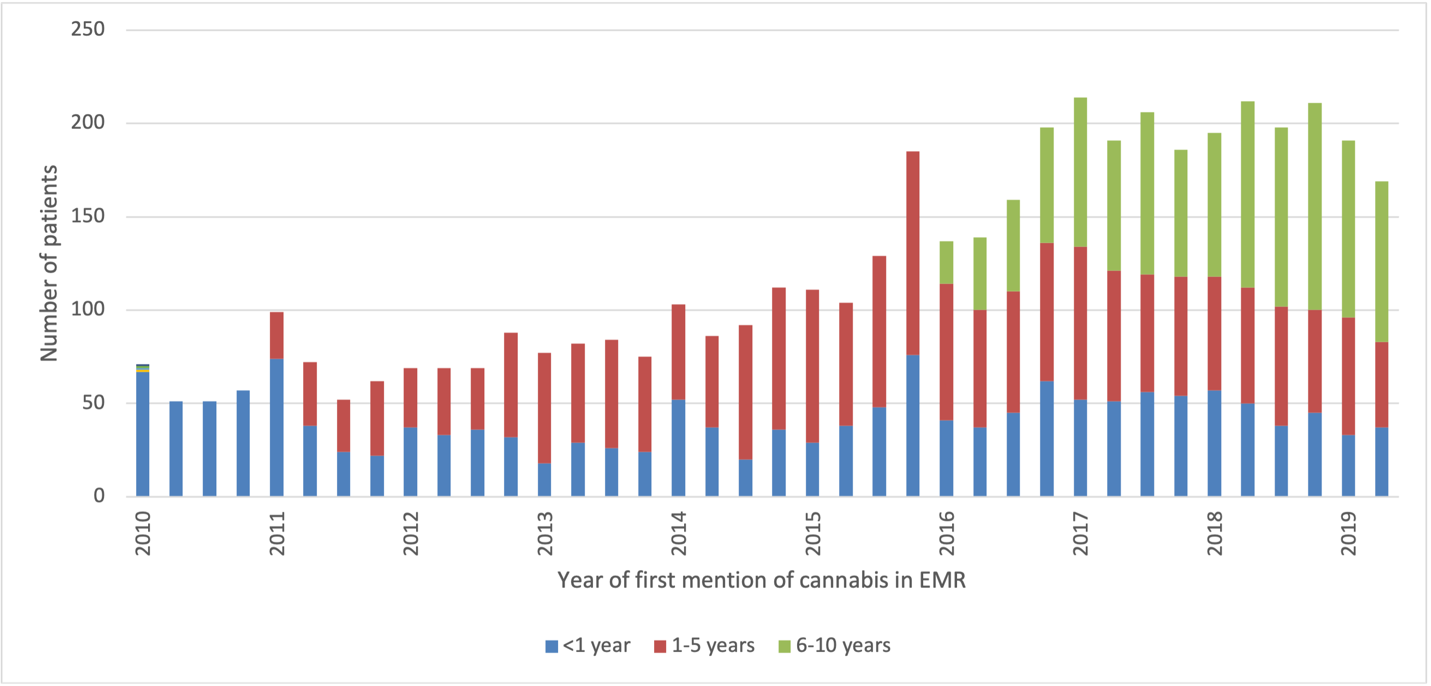

Supplement: Supplementary file 1 — Additional file 1: Figure S1. Length of time to the first mention of cannabis in the patient record (from earliest date in the EMR). [file 13104_2023_6274_MOESM1_ESM.docx]
