# Supplementary material for: Documenting cannabis use in primary care: a descriptive cross-sectional study using electronic medical record data in Alberta, Canada
Source: BMC Res Notes. 2023 Feb 1;16:9. doi: 10.1186/s13104-023-06274-6 (PMC9890680; doi:10.1186/s13104-023-06274-6)
Supplement: Supplementary file 2 — Additional file 2: Table S1. Search terms used in the EMR data. [file 13104_2023_6274_MOESM2_ESM.docx]

**Table S1.** Search terms used in the EMR data.

| **Code or text match^1^** | **Required inclusion text^2^** | **Exclusion text^3^** | **Rationale** |
| --- | --- | --- | --- |
| 304.3 |  |  | ICD-9 code for cannabis dependence |
| 305.2 |  |  | ICD-9 code for non-dependent cannabis abuse |
| N02BG10 |  |  | ATC code for cannabinoids (including nabiximols) |
| CANN[A-Z]B |  |  | Cannabis and related spelling variations |
| CBD |  | OBSTRUCT  STONE  DILAT  STENT  CHOLE  DISTEND  ERCP  ENLARG  STRICT  BLOCK  CT  FLAR | Includes CBD in the context of cannabidiol  Excludes records related to the common bile duct, abbreviated CBD |
| HASH |  |  | Hash or hashish |
| MARIJ |  |  | Marijuana and related spelling variations |
| MARIH |  |  | Marihuana and related spelling variations |
| MARINOL |  |  | Medical marijuana |
| MJ |  |  | Common slang term for marijuana |
| NABILONE |  |  | Medical marijuana |
| POT | USE  USING  TRIED  USED | NET.*^4^ | Exclude records related to Neti pots (and related spelling variations) |
| POT | SMOK  DRUG  ADDICT  QUIT  HIGH  ABUSE  OCC  CIG  ALCOHOL |  | Include all records of “pot” in the context of drug use |
| SATIVA |  |  | Type of cannabis used for medicinal purposes |
| SATIVEX |  |  | Medical marijuana |
| THC |  |  | Abbreviation for tetrahydrocannabinol |
| WEED |  | ALLERG  RAG  POLLEN  GRASS | Exclude records for allergies (e.g., ragweed) |
| Bud, CBG, dope, ganja, grass, hemp, herb, indica, kush, ‘mary jane’, spliff |  |  | These terms were considered and tested in the search, but ultimately excluded due to an absence of records or a high proportion of unrelated records. |

^1^ Cannabis related search term. ‘[A-Z]’ indicates a match with a single letter from A to Z.
^2^ At least one of the listed terms in the inclusion must be in the EMR record for the entry to be considered relevant to cannabis.
^3^ A match with any of the listed the terms exempts the EMR record from being considered cannabis-related.
^4^ ‘.*’ indicates a match for zero or more occurrences of any character, excluding new lines
