# Supplementary material for: Documenting cannabis use in primary care: a descriptive cross-sectional study using electronic medical record data in Alberta, Canada
Source: BMC Res Notes. 2023 Feb 1;16:9. doi: 10.1186/s13104-023-06274-6 (PMC9890680; doi:10.1186/s13104-023-06274-6)
Supplement: Supplementary file 3 — Additional file 3: Table S2. Differences in cannabis-related recording by type of EMR system. [file 13104_2023_6274_MOESM3_ESM.docx]

**Table S2.** Differences in cannabis-related recording by type of EMR system.

| **Type of EMR System** | Number of clinics and patients contributing to CPCSSN in Alberta | Providers with at least one cannabis record for their patients, n (%) | Patient with at least one record of cannabis use in their EMR, n (%) |
| --- | --- | --- | --- |
| Wolf | Clinics: 25  Patients: 169 097 | 153 (98.1) | 2 109 (1.2) |
| Med Access | Clinics: 25  Patients: 194 517 | 150 (96.8) | 1 975 (1.0) |
| Accuro | Clinics: 2  Patients: 12 040 | 8 (88.9) | 205 (1.7) |
| Healthquest | Clinics: 1  Patients: 4 726 | 3 (100.0) | 49 (1.0) |
| Practice Solutions | Clinics: 2  Patients: 18 150 | 9 (90.0) | 314 (1.7) |
|  |  | p=0.348 | p<0.001 |
