# Supplementary material for: Documenting cannabis use in primary care: a descriptive cross-sectional study using electronic medical record data in Alberta, Canada
Source: BMC Res Notes. 2023 Feb 1;16:9. doi: 10.1186/s13104-023-06274-6 (PMC9890680; doi:10.1186/s13104-023-06274-6)
Supplement: Supplementary file 4 — Additional file 4: Table S3. Characteristics of patients with a cannabis term recorded in their primary care electronic medical record, stratified by presence of record in the Medication table. [file 13104_2023_6274_MOESM4_ESM.docx]

**Table S3.** Characteristics of patients with a cannabis term recorded in their primary care electronic medical record, stratified by presence of record in the Medication table.

| **Characteristics of Patients with Cannabis Record** | **Record in Medication Table**,  n (%)  N = 1 644 | **No Record in Medication Table**, n (%)  N = 3 008 | P-Value |
| --- | --- | --- | --- |
| Sex | | | <0.001 |
| Male | 802 (48.8) | 1 859 (61.8) |  |
| Female | 842 (51.2) | 1 148 (38.2) |  |
| Other | 0 (0.0) | 1 (<0.1) |  |
| Age group in years | | | <0.001 |
| Under 10 | 3 (0.2) | 9 (0.3) |  |
| 10-19 | 6 (0.4) | 150 (5.0) |  |
| 20-29 | 57 (3.5) | 806 (26.8) |  |
| 30-39 | 180 (10.9) | 702 (23.3) |  |
| 40-49 | 291 (17.7) | 474 (15.8) |  |
| 50-59 | 461 (28.0) | 473 (15.7) |  |
| 60-69 | 383 (23.3) | 300 (10.0) |  |
| 70 and older | 263 (16.0) | 94 (3.1) |  |
| Residence location | | | <0.001 |
| Rural | 376 (22.9) | 396 (13.2) |  |
| Urban | 1 197 (72.8) | 2 332 (77.5) |  |
| Missing postal code | 71 (4.3) | 280 (9.3) |  |
| Presence of chronic conditions as defined by CPCSSN algorithms | | | |
| CKD | 169 (10.3) | 50 (1.7) | <0.001 |
| COPD | 337 (20.5) | 251 (8.3) | <0.001 |
| Depression | 1 075 (65.4) | 1 591 (52.9) | <0.001 |
| Diabetes mellitus | 363 (22.1) | 193 (6.4) | <0.001 |
| Epilepsy | 83 (5.0) | 120 (4.0) | 0.106 |
| Hypertension | 734 (44.6) | 453 (15.1) | <0.001 |
| Osteoarthritis | 613 (37.3) | 323 (10.7) | <0.001 |
| Parkinson’s disease | 20 (1.2) | 11 (0.4) | 0.001 |
| Pediatric asthma | 13 (0.8) | 134 (4.5) | <0.001 |
| None* | 149 (9.1) | 955 (31.7) | <0.001 |

*no co-morbid conditions from 13 conditions that have a CPCSSN validated case definition
